# Costs and Its Determinants in Pituitary Tumour Surgery

**DOI:** 10.3389/fendo.2022.905019

**Published:** 2022-07-07

**Authors:** Alies J. Dekkers, Friso de Vries, Amir H. Zamanipoor Najafabadi, Emmy M. van der Hoeven, Marco J. T. Verstegen, Alberto M. Pereira, Wouter R. van Furth, Nienke R. Biermasz

**Affiliations:** ^1^Department of Medicine, Division of Endocrinology, Pituitary Center and Center for Endocrine Tumors, Leiden University Medical Center, Leiden, Netherlands; ^2^Department of Medicine, Center for Endocrine Tumours Leiden, Leiden University Medical Center, Leiden, Netherlands; ^3^Department of Neurosurgery, Leiden University Medical Center, University Neurosurgical Center Holland, Leiden, Netherlands; ^4^Directorate of Finances, Leiden University Medical Center, Leiden, Netherlands; ^5^Department of Endocrinology and Metabolism, Amsterdam University Medical Center, Meibergdreef 9, Amsterdam, Netherlands

**Keywords:** pituitary tumour, pituitary adenoma, pituitary surgery, value-based healthcare, cost analysis, transsphenoidal surgery

## Abstract

**Purpose:**

Value-based healthcare (VBHC) provides a framework to improve care by improving patient outcomes and reducing healthcare costs. To support value-based decision making in clinical practice we evaluated healthcare costs and cost drivers in perioperative care for pituitary tumour patients.

**Methods:**

We retrospectively assessed financial and clinical data for surgical treatment up to the first year after surgery of pituitary tumour patients treated between 2015 and 2018 in a Dutch tertiary referral centre. Multivariable regression analyses were performed to identify determinants of higher costs.

**Results:**

271 patients who underwent surgery were included. Mean total costs (SD) were €16339 (13573) per patient, with the following cost determinants: surgery time (€62 per minute; 95% CI: 50, 74), length of stay (€1331 per day; 95% CI 1139, 1523), admission to higher care unit (€12154 in total; 95% CI 6413, 17895), emergency surgery (€10363 higher than elective surgery; 95% CI: 1422, 19305) and postoperative cerebrospinal fluid leak (€14232; 95% CI 9667, 18797). Intradural (€7128; 95% CI 10421, 23836) and combined transsphenoidal/transcranial surgery (B: 38494; 95% CI 29191, 47797) were associated with higher costs than standard. Further, higher costs were found in these baseline conditions: Rathke’s cleft cyst (€9201 higher than non-functioning adenoma; 95% CI 1173, 17230), giant adenoma (€19106 higher than microadenoma; 95% CI 12336, 25877), third ventricle invasion (€14613; 95% CI 7613, 21613) and dependent functional status (€12231; 95% CI 3985, 20477). In patients with uncomplicated course, costs were €8879 (3210) and with complications €17551 (14250).

**Conclusions:**

Length of hospital stay, and complications are the main drivers of costs in perioperative pituitary tumour healthcare as were some baseline features, e.g. larger tumors, cysts and dependent functional status. Costs analysis may correspond with healthcare resource utilization and guide further individualized care path development and capacity planning.

## 1 Introduction

Clinically apparent pituitary adenomas are rare with an incidence of 3.9-7.4 persons per 100.000 per year and a prevalence of 1 per 1000 persons (1). Patients present with a variety of symptoms due to mass effects (e.g., chiasm compression, hypopituitarism) and/or hormonal hypersecretion, depending on the adenoma subtype. Preferred treatment in most cases is transsphenoidal resection (2–4), with or without a combination of medical therapy. Radiotherapy is used as a last resort in selective cases.

There are disease-specific treatment guidelines (2, 3, 5–7), however, in clinical practice the evidence base of optimal management choices is rather limited. Patients show highly variable clinical outcomes and health-related quality of life after treatment (8, 9). The Leiden University Medical Centre aims to improve pituitary tumour healthcare by adapting and implementing a value-based healthcare (VBHC) approach with prospective real-time outcome evaluations. VBHC demonstrates a framework to restructure healthcare with the ultimate goal of improving outcomes and/or reducing (unnecessary) costs, and thus increase value for patients (10, 11). Accordingly, our team designed and published a value-based framework to measure perioperative outcomes in pituitary tumour patients, allowing clinicians to evaluate and individualize healthcare (8). Subsequently, we expect that assessment of healthcare costs, which reflects in-hospital resource utilization, results in development of cost reduction initiatives and better individualized care pathways, while maintaining or improving clinical outcomes.

A comprehensive cost evaluation of perioperative care with extensive analyses of cost drivers for pituitary tumour patients is currently lacking. Previous cost evaluation studies in pituitary healthcare were limited to the surgical procedure (12), index hospitalization (12–14), complications or readmissions (15, 16), instead of encompassing the entire perioperative trajectory. Other studies focussed on a single diagnosis, limiting comparisons between pituitary tumour types and other case mix variables and prohibiting evaluation of the care pathways and the required capacity within the pituitary healthcare team (17–29). Moreover, many studies assessed predictors of healthcare costs instead of cost determinants (24, 28) with predictors not being cost drivers *per se*. Therefore, this study aims to evaluate 1) total in-hospital costs during pituitary tumour surgery, and up to the first year after; 2) how total costs are attributable to different cost domains (i.e., costs for surgery, hospitalization, irradiation and diagnostic investigations); and 3) which determinants are drivers of total costs. Based on our results and prior knowledge we will propose strategies for improving VBHC care pathways.

## 2 Materials and Methods

### 2.1 Study Design

This retrospective study was performed using data of consecutive surgically treated pituitary tumour patients between January 2015 and December 2018 at the Leiden University Medical Centre (LUMC), a tertiary referral centre for pituitary surgery in the Netherlands. Patients were eligible if diagnosed with a pituitary adenoma, Rathke’s cleft cyst (RCC) or craniopharyngioma. Patients with other tumours in the (para)sellar region (e.g., meningioma, cerebral metastases, chordoma, and chondrosarcoma) or uncertain diagnosis were excluded. Clinical data were collected from electronic patient charts and in-hospital costs data were extracted from a financial database constructed for VBHC research. Data of patients were obtained after approval of the scientific committee of the department, after which a waiver was obtained from the institution medical ethics committee, local study number (G19·011).

### 2.2 Perioperative Care Trajectory

Preoperative evaluation of all patients includes imaging (MRI and CT-scan), comprehensive endocrine work-up of all pituitary axes, and a neuro-ophthalmological assessment when indicated. During a combined consultation with an endocrinologist and neurosurgeon, the main indication for surgery (e.g., hypersecretion, visual symptoms), aim of surgery (e.g., complete resection, gross total resection (GTR), chiasm decompression) and intended effect (e.g. biochemical remission, recovery of visual function, lowering medication dosage) are established, depending on the patient’s symptoms and surgical feasibility of pituitary tumour resection. Based on last preoperative imaging all pituitary tumours, including RCC’s and craniopharyngioma’s, were classified as micro- (<10mm), macro- (10-40mm) or giant (>40mm) adenoma for comparability. Third ventricle invasion was established and cavernous sinus invasion was defined as a KNOSP-score of ≥3 (30). Patient characteristics and comorbidities were determined during hospital admission. Hormonal hypersecretion and hypopituitarism are defined according to current guidelines and hormone replacement therapy was initiated when indicated, especially in case of corticotropic or thyrotropic insufficiency [8, 16, 33, 34]. Prior treatment with pharmacotherapy (e.g., cabergoline, metyrapone, octreotide), surgery, or irradiation was established.

Endoscopic transsphenoidal surgery is generally performed by two neurosurgeons and open microscopic transcranial surgery by one surgeon. Patients with complex nasal anatomy or extensive skull base destruction are operated together with an ENT-surgeon. Most surgical resections were performed endoscopically transsphenoidal. When necessary, an extended or intradural approach was performed. In some cases of giant adenomas or post-operative apoplexy in a suprasellar adenoma remnant, a craniotomy or combined endoscopic transsphenoidal and microscopic transcranial surgery was performed. We described our surgical approach in detail in a recent review (31).

Following surgery, patients were discharged at postoperative day 2 or 3 when eligible for a short-stay protocol, instead of at postoperative day 5 (32). Length of stay (LOS) was measured from the day of surgery until discharge. Admission to a high care unit was defined as hospitalization at the medium- or intensive care unit. Subsequently, HPA-axis function was evaluated within one week after surgery through fasting cortisol or dynamic testing (33). Patients were daily monitored by a case manager for occurrence of complications in the first 2 weeks postoperatively and in case of postoperative hyponatraemia readmission was considered If fluid restriction in the home situation was not sufficient. Diabetes insipidus (DI) was evaluated and classified based on duration. Accordingly, patients with DI lasting for shorter or longer than 2 weeks were classified as 1-2 or 3-4, respectively (34). An outpatient clinic visit for evaluation of treatment outcomes was performed at 6 months, or sooner in case of complications or complaints, which consists of evaluation of residual tumour on MRI, ophthalmological examination (visual acuity and visual fields testing through static perimetry), and assessment of hormone levels. Remission of hormonal hypersecretion, hypopituitarism, and vision were defined according to guidelines and recent literature (5–7, 35, 36).

### 2.3 Costs Analyses

Our costs analyses adhere to the Consolidated Health Economic Evaluation Report Standards (37). Direct costs were derived from an institutional perspective, conform the most recent national guidelines for in-hospital cost assessment (38). Costs are displayed in euros and can be converted into American dollars by applying the mean purchasing power parity (PPP), as reported by the Organisation for Economic Co-operation and Development (OECD). The mean PPP between 2015 and 2018 was 0,791 per dollar (39). The reported costs are not corrected for inflation and a 0% discount rate was applied. In line with the study’s perspective, we calculated direct cost as opposed to indirect costs, because they are directly related to healthcare utilization and so a reflection of care capacity. The costs were attributed to units using cost allocation keys, meaning that costs for resources were activity-adjusted, e.g., costs for surgery or consultations were calculated based on the estimated mean duration of the activity.

Total costs during follow-up and the distribution of total costs into cost domains (i.e., costs for surgery, hospitalization, irradiation and diagnostic investigations or consultations) were established for the total cohort. Surgical costs were based on costs for resources during the surgical procedure, including surgery materials, surgeons, nurses and monitoring devices. Hospitalization costs comprised expenditures for the general ward, ICU, physician visits and nurses, including readmissions. Diagnostic investigations or consultations costs consisted of laboratory tests, radiology, pathology and (para)medic consultations. Prescribed medication at our hospital and expenditures at other facilities than the LUMC, such as the general practitioner, were not included.

### 2.4 Statistical Analyses

Data analyses were performed using the SPSS Statistics Version 25. For categorical variables, the number of patients and corresponding percentage of the total population were calculated. For continuous variables, the mean with range and standard deviation are reported. Missing data were excluded from analyses using pairwise deletion.

First of all we report the mean total costs and how these costs are distributed amongst different cost domains (i.e., surgery, hospitalization, irradiation, diagnostic investigations or consultations) of the total cohort. For each cost domain, both absolute cost data and percentage of total costs are reported. Subsequently, univariable and multivariable linear regression analyses were performed to identify factors associated with higher costs. For each assessed determinant, a separate multivariable analysis was performed, estimating the association between the determinant of interest and total costs, corrected for the relevant confounders. Confounders were defined as variables associated with both the determinant and the outcomes, but not being caused by the determinant, i.e., not laying within the causal path between the determinant and outcomes (40). Confounders were chosen for each determinant following this definition and using prior clinical knowledge. The effect sizes of the regression analyses are reported in unstandardized regression coefficients and 95% confidence intervals (95% CI) (41), with P-values lower than 0,05 considered significant.

Furthermore, we analyse how total costs of determinants were distributed amongst different cost domains, expecting that some determinants would increase costs among a specific cost domain. For example, larger tumour size may require prolonged, complex surgery and therefore increased surgery costs may the main cost driver. Complications may prolong length of stay and mainly increase hospitalization costs. By analysing how each cost domain contributes to total costs we aim to investigate whether increased total costs are explained by specific parts of the healthcare pathways, which may serve as target for future value-based healthcare initiatives.

## 3 Results

### 3.1 Study Population

**Figure 1:**
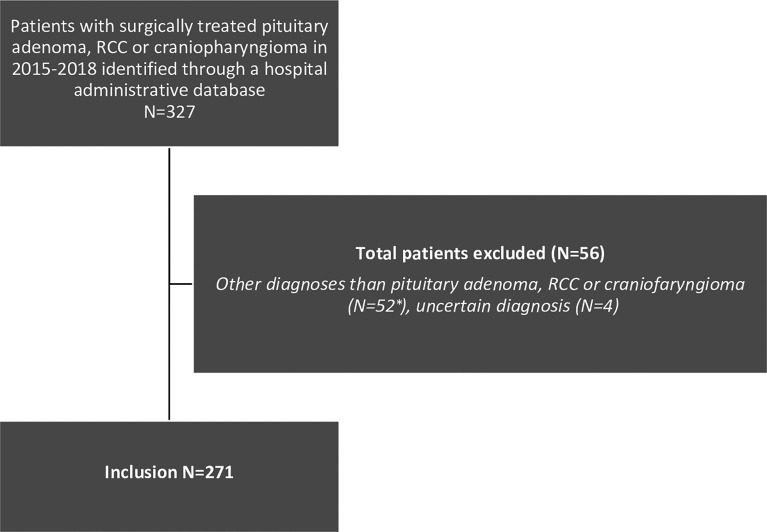
Flow chart of patients’ selection. * Excluded diagnoses: meningioma (N=31), metastasis (N=5), arachnoid cyst (N=1), chordoma (N=5) chondrosarcoma (N=3), Schwannoma (N=2), glioblastoma (N=1), medulloblastoma (N=1), giant cell tumour (N=1), plasmacytoma (N=1), pituicytoma (N=1).

A total of 271 patients with a pituitary tumour met the inclusion criteria (**Figure 1**). Demographics, clinical characteristics, perioperative factors and outcomes are displayed in **Table 1**. Mean (SD) age was 50 (17) years, 147 patients were female (54%), mean BMI was 27,6 (4,9) and 18% were currently smoking. Patients were diagnosed with non-functioning adenoma (NFA) (n=118; 44%), acromegaly (n=42; 16%), prolactinoma (n=39; 13%), Cushing’s disease (n=36; 13%) craniopharyngioma (n=20; 7%), RCC (n=10; 4%) FSH-adenoma (n=3; 1%) or thyrotropinoma (n=3; 1%). The majority of patients had a macroadenoma (n=188; 69%), 51 (19%)a microadenoma, 21 (8% a giant adenoma and in 11 (4%) of patients no clear adenoma was visible. Comorbidities included hypertension (n=80; 30%) diabetes mellitus (n=24; 9%), COPD (n=3; 1%) and congestive heart failure (n=1; <1%), and 11 (4%) patients were functionally dependent. Based on these comorbidities, the mFI-5 score of patients were 0 (n=174; 64%), 1 (n=79; 29%), 2 (n=15; 6%), 3 (n=2; 1%) or 4 (n=1; <1%). Prior to surgery, 28 (11%) had cortisol deficiency, 73 (27%) had hypopituitarism amongst other axes and 37 (14%) had panhypopituitarism. Vision loss or VFD was established in 116 (44%) and a cranial nerve deficit in 18 (7%). The tumour invaded the third ventricle or cavernous sinus in 17 (7%) and 46 (17%) of patients, respectively. In 27 (10%) apoplexy was determined. Some patients had undergone prior treatment; 89 patients (33%) had received pharmacological treatment, 52 patients (19%) had previous surgery and 2 patients (1%) received prior radiotherapy.

**Table 1:** Baseline characteristics displayed in number of patients with corresponding percentage or mean with standard deviation.

Preoperative factors*Demography, disease characteristics and comorbidities*			Surgery related factors*Surgery indication and approach*			Post-surgery*Complications, outcomes and costs*		
**Variable**	**Category**	**N (%)**	**Variable**	**Category**	**N (%)**	**Variable**	**Category**	**N (%)**
**Sex**	Male	124 (46%)	**Surgical procedure**	Endoscopic transsphenoidal adenectomy	260 (96%)	**Complications**	Intracerebral haemorrhage or hematoma requiring intervention	9 (3%)
	Female	147 (54%)		Craniotomy	2 (<1%)		Postoperative CSF leak	26 (10%)
Age (years)*		50 (17)		Endoscopic transventricular	2 (<1%)		Hyponatremia (including SIADH)	48 (18%)
BMI*		27,6 (4,9)		Combined endoscopic transsphenoidal and microscopic transcranial	7 (3%)		Hypothalamic injury	1 (<1%)
**Currently smoking**		47 (18%)	**Elective or emergency surgery**	Elective	248 (92%)		Thromboembolic event	6 (2%)
**Clinical diagnosis**	NFA	118 (44%)		Emergency	23 (8%)		Epistaxis requiring intervention	19 (7%)
	Acromegaly	42 (16%)	**Surgery indication**	Prevention of disease progression	18 (7%)		New hypopituitarism (excl. cortisol deficiency)	1 (<1%)
	Cushing’s disease	36 (13%)		Hypersecretion	110 (41%)		New cortisol deficiency	8 (3%)
	Prolactinoma	39 (13%)		Growth of tumour residual	10 (4%)		New panhypopituitarism	8 (3%)
	Thyrotropinoma	3 (1%)		Apoplexy	18 (7%)		Vision deterioration	2 (<1%)
	FSH-adenoma	3 (1%)		VFD	60 (22%)		New cranial nerve palsy	0 (0%)
	Craniopharyngioma	20 (7%)		Vision	41 (15%	**Diabetes insipidus classification**	0: no DI	181 (67%)
	RCC	10 (4%)		Hydrocephalus	4 (2%)		1: transient DI <48 hours, spontaneously resolving without treatment	23 (8%)
**Tumour size**	Microadenoma	51 (19%)		CSF leak	2 (<1%)		2: transient DI <2 weeks	27 (10%)
	Macroadenoma	188 (69%)		Cranial nerve deficit	2 (<1%)		3: DI between 2 weeks and 6 months	22 (8%)
	Giant adenoma	21 (8%)		Headache	3 (1%)		4: persistent DI >6 months	18 (7%)
	No clear adenoma	11 (4%)		Nose congestion	1 (<1%)	**CSF leak treatment** (N=26)	ELD	6 (23%)
**Comorbidities**	Hypertension	80 (30%)		Behavioural alterations	1 (<1%)		Surgery	12 (46%)
	Diabetes Mellitus	24 (9%)		Biopsy	1 (<1%)		ELD and surgery	8 (31%)
	Congestive heart failure	1 (<1%)	**Surgery aim**	Gross-total resection	109 (40%)	LOS of index hospitalization in days*		5,7 (6,6)
	COPD	3 (1%)		Decompression	107 (40%)	**Admission at high care unit during index hospitalization**		18 (7%)
	Functional status	11 (4%)		Emergency	23 (9%)	LOS at high care unit during index hospitalization in days* (N=18)		2,2 (2,3)
**mFI-5 score**	0	174 (64%)		Exploration	17 (6%)	**Emergency department visit <30 days**		35 (13%)
	1	79 (29%)		Debulking	13 (5%)	**Readmission <30 days**		41 (15%)
	2	15 (6%)		Biopsy	2 (<1%)	**Recovery 6 months after surgery**		
	3	2 (1%)	**Surgery intended effect**	Curative	100 (37%)			
	4	1 (<1%)		Recovery VFD	60 (22%)	**Visible tumour residual on MRI** (N=255)		108 (42%)
	5	0 (0%)		Recovery vision	40 (15%)	**Recovery hypersecretion** (N=124)	Yes	82 (66%)
**Hypopituitarism (N=268)**	Hypopituitarism non-corticotrope	73 (27%)		Protect vision	28 (10%)		Partial	14 (11%)
	Cortisol deficiency	28 (11%)		Decrease sudden headache/vision loss	18 (7%)		No	28 (23%)
	Panhypopituitarism	37 (14%)		Reduce medication dosage	10 (4%)	**Recovery hypopituitarism** (N=134)	Yes	29 (22%)
**Vision loss or VFD (N=265)**		116 (44%)		Recovery hydrocephalus	4 (2%)		Partial	11 (8%)
**Cranial nerve deficit**		18 (7%)		Recover cranial nerve function	2 (<1%)		No	94 (70%)
**Invasion third ventricle** **(N=253)**		17 (7%)		Recover CSF leak	2 (<1%)	**Recovery vision loss** (N=66)	Yes	51 (77%)
**Invasion cavernous sinus**		46 (17%)		Protect pituitary function	1 (<1%)		Partial	10 (15%)
**Apoplexy**		27 (10%)		PA diagnosis	2 (<1%)		No	5 (8%)
**Previous treatment received**	Pharmacological	89 (33%)		Improve nose congestion	1 (<1%)	**Recovery VFD deficit** (N= 114)	Yes	80 (70%)
	Surgery	52 (19%)		Enable irradiation	1 (<1%)		Partial	28 (25%)
	Radiation	2 (<1%)		Reduce headache	2 (<1%)		No	6 (9%)
**Year of surgery**	2015	60 (22%)	OR time in minutes*		188,8 (129,8)	**Costs in euros**		
	2016	62 (23%)	**Extended approach**		22 (8%)	Total costs*		16339 (13573)
	2017	67 (25%)	**Intradural surgery**		16 (6%)	Surgery costs*		8979 (8523)
	2018	82 (30%)	**Surgery performed by two neurosurgeons and ENT-surgeon**		43 (16%)	Hospitalization costs*		4568 (5039)
			**Intra-operative CSF leak**		94 (35%)	Irradiation costs* (N=9)		283 (332)
			**Reconstruction with nasoseptal flap**		43 (16%)	**Diagnostic investigations and consultations costs**		2783 (1584)

*Mean with range and standard deviation.

N, number; SD, standard deviation; BMI, body mass index; NFA, non-functioning adenoma; FSH, follicle stimulating hormone; RCC, Rathke’s cleft cyst; COPD, chronic obstructive pulmonary disease; mFI, modified frailty index; VFD, visual field deficit; CSF, cerebrospinal fluid; GTR gross total resection; OR, operating room; ENT, ear, nose and throat; DI, diabetes insipidus; SIADH, syndrome of inappropriate antidiuretic hormone secretion; ELD, external lumbar drain; LOS, length of stay.

The indication of surgery was hormonal hypersecretion (n=110; 41), VFD (n=60; 22%), vision loss (n=41; 15%), apoplexy (n=18; 7%), prevention of disease progression (n=18; 7%), growth of tumour residual (n=10; 4%), hydrocephalus (n=4; 2%), headache (n=3; 1%), CSF leak (n=2; <1%), cranial nerve deficit (n=2; <1%), nose congestion (n=1; <1%), behavioural alterations (n=1; <1%) or to perform a biopsy (n=1; <1%). The surgical aim was GTR (n=109; 40%), decompression (n=107; 40%), emergency (n=23, 9%), exploration (n=17; 6%), debulking (n=13; 5%) or biopsy (n=2; <1%). The intended effect was curative or remission (n=100; 37%), recover VFD (n=66; 22%), recover vision loss (n=40; 15%) prevent vision loss (n=18; 7%), reduce mediation dosage (n=10; 4%), recover cranial nerve function (n=2; <1%), recover CSF leak (n=2; <1%), protect pituitary function (n=1; <1%), obtain histopathological diagnosis (n=2; <1%), improve nose congestion (n=1; <1%) or reduce headache.

Patients underwent surgery in 2018 (n=82; 30%), 2017 (n=67; 25%), 2016 (n=62; 23%) or 2015 (n=60; 22%) on an elective basis (n=248; 92%) or as an emergency (n=23; 8%) with a mean surgery duration of 189 (130) minutes. Endoscopic trans-sphenoidal surgery was performed in 260 (96%) patients, of which 22 patients (8%) underwent extended endoscopic surgery and in 16 cases (6%) intradural trans-sphenoidal surgery was performed. Other surgical procedures included endoscopic transventricular (n=2; 1%), transcranial (n=2; 1%) and combined endoscopic transsphenoidal/microscopic transcranial surgery (n=7; 3%). Most procedures were performed by two neurosurgeons, in 43 (16%) surgery was performed together with an ENT-surgeon. Intra-operative CSF leak occurred in 94 (35%) and in 43 (16%) skull base reconstruction with nasoseptal flap was performed.

Mean length of stay (LOS) was 6 (7) days. 18 patients (7%) were admitted to the medium or intensive care (IC) unit during index hospitalization with a mean LOS of 2 (2) days. Postoperative complications were hyponatremia or SIADH (n=48; 18%), CSF leak (n=26; 10%), epistaxis (n=19; 7%), intracerebral haemorrhage or hematoma (n=9; 3%), cortisol deficiency (n=8; 3%), panhypopituitarism (n=8; 3%), thromboembolic event (n=6; 2%), vision deterioration (n=2; <1%), hypothalamic injury (n=1; <1%) or other (i.e., no cortisol deficiency) hypopituitarism (n=1; <1%). Diabetes insipidus (DI) occurred in 90 (33%) and resolved within 48 hours (DI 1; n=23; 8%), 2 weeks (DI 2; n=27; 10%) or 6 months (DI 3; n=22; 8%). In 18 (7%) DI persisted for over 6 months (DI 4). CSF leak was treated with surgery (n=12; 46%), ELD (n=6; 23%) or both ELD and surgery (n=8; 31%).

### 3.2 Healthcare Costs

**Figure 2:**
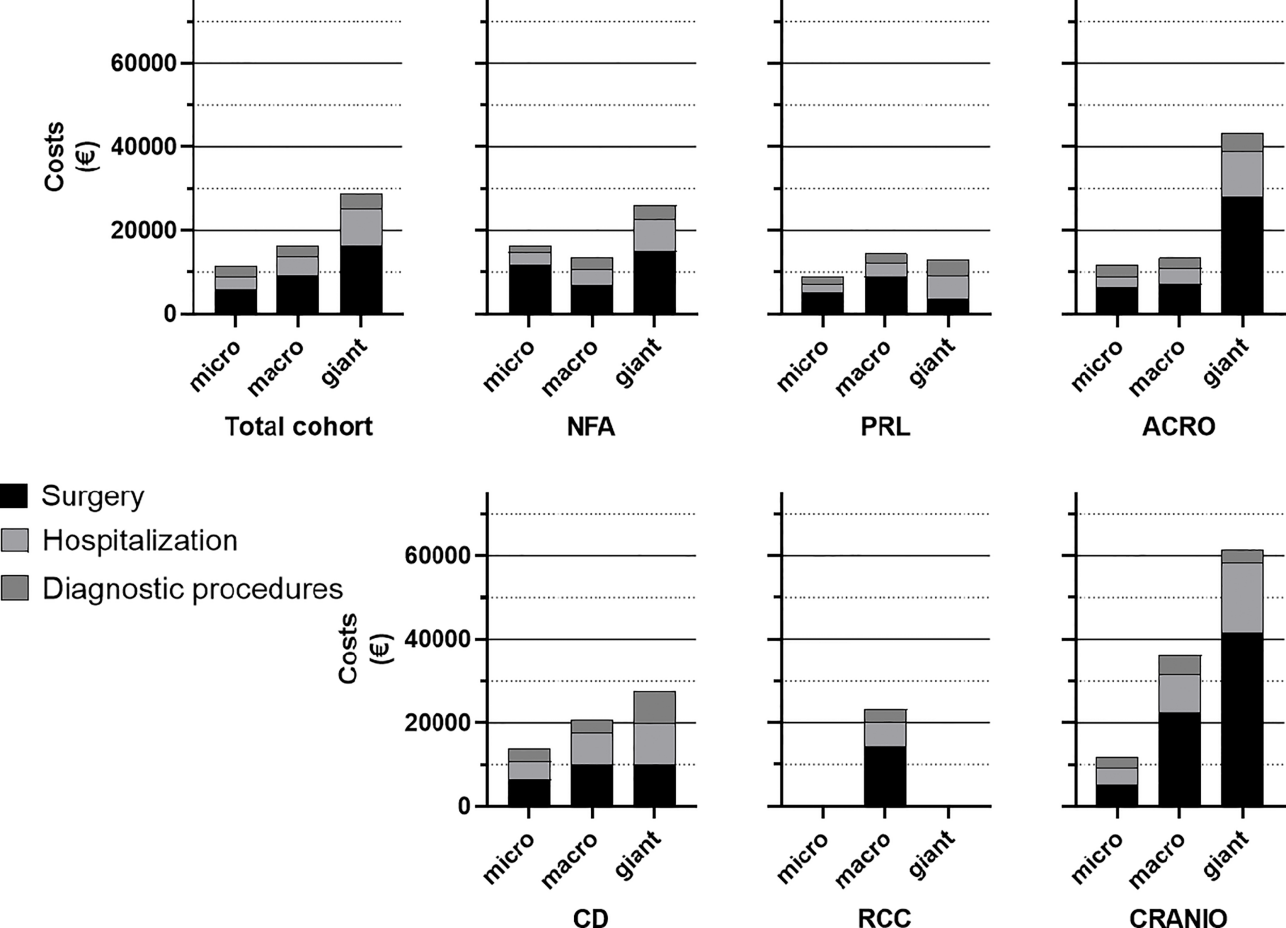
Stacked bars displaying mean total costs of patients with different diagnoses and tumour size. For comparability, tumour size of patients with RCC or craniopharyngioma were also categorized as micro, macro or giant tumours. In most groups, surgery costs account for >50% of total costs and total costs increase with tumour size. *NFA*, non-functioning adenoma; *PRL*, Prolactinoma; *ACRO*, acromegaly; *CD*, Cushing’s disease; *RCC*, Rathke’s cleft cyst; *CRANIO*, craniopharyngioma.

Mean total costs were €16339 (13573) per patient for surgery and the first-year post-operative care. Total costs comprised mean costs for surgery (€8979, SD 8523), hospitalization (€4568, SD 5039), irradiation (n=9; €283, SD 332) and diagnostic investigations or consultations (€2783, SD 1584). For the total cohort, costs for surgical care account for 55% of the total costs, hospitalizations for 28%, diagnostic investigations or consultations for 17%. Costs for irradiation comprise less than 1% of total costs with only few (n=9) subjected to radiotherapy in this study period (data not shown). Costs stratified for different pituitary tumour diagnoses and tumour size are presented in **Figure 2**. Mean costs (SD) for patients with pituitary adenoma are €14861 (11962) for NFA, €11560 (5824) for prolactinoma, €14496 (3471) for FSH-adenoma, €8649 (1566) for thyrotropinoma, €14973 (11556) for acromegaly and €15868 (8055) for Cushing’s disease. Mean total costs for patients with RCC or craniopharyngioma are €23263 (24788) and €36129 (21388), respectively. Mean total costs separated per tumour size are €11344 (5513) for microadenomas, €16478 (12383) for macroadenomas and €28707 (26926) for giant adenomas. Mean costs for patients with no clear adenoma on MRI (comprising patients with Cushing’s disease) are €13516 (6875).

### 3.3 Determinants of Total Costs

**Figure 3:**
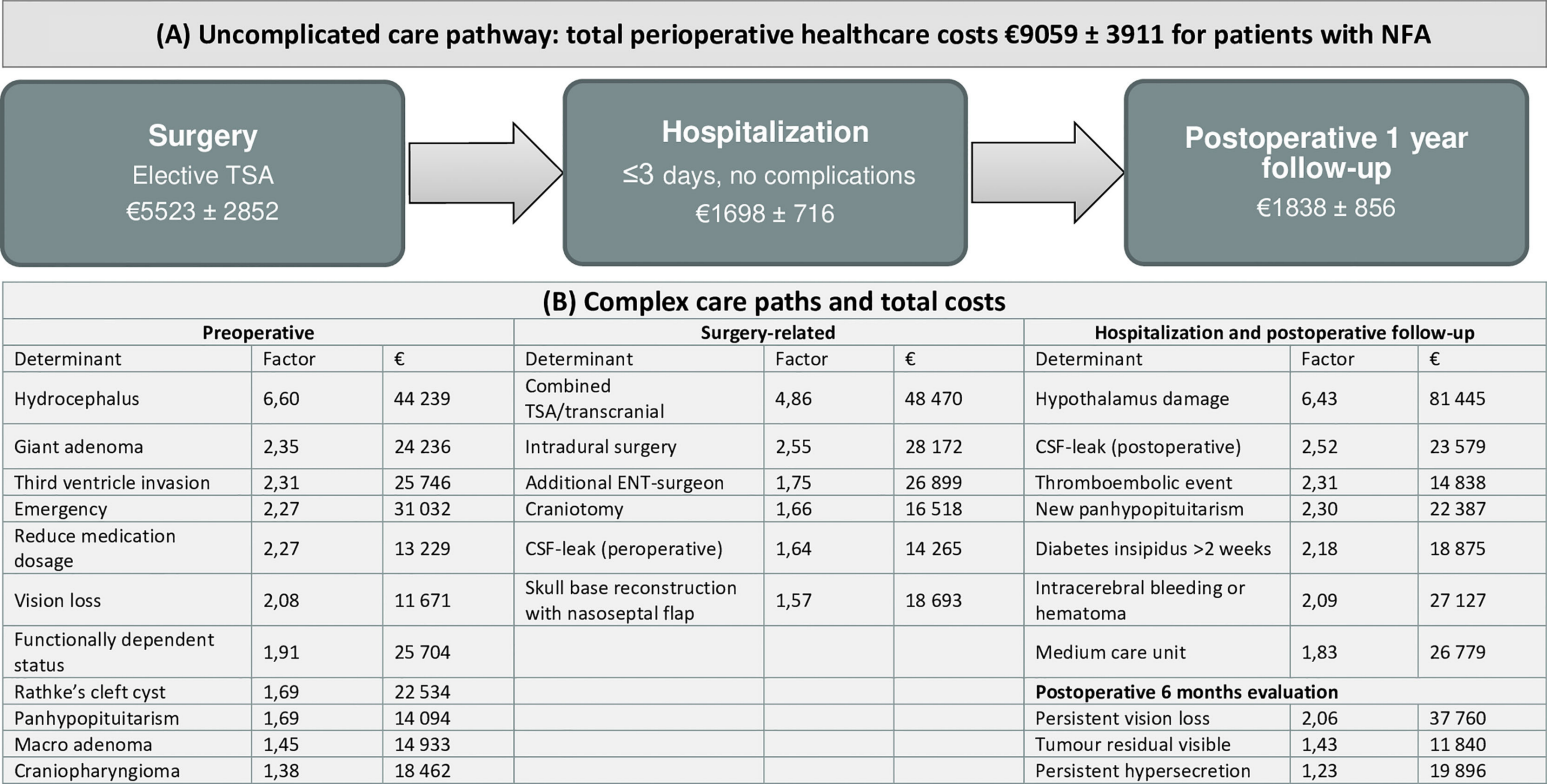
Overview of perioperative healthcare costs for patients undergoing pituitary surgery. **(A)** To illustrate, the care trajectory of patients with non-functioning micro- or macroadenoma undergoing transsphenoidal adenectomy is shown as an example for an uneventful case. **(B)** Cost determinants are summarized in coefficients (calculated by dividing total costs of the determinant by total costs of the reference group) and total perioperative costs, based on multivariable analyses (**Supplemental Table S1**) E.g., total healthcare costs for patients with postoperative CSF leak is 2,52 times higher compared to patients without CSF leak. NFA, non-functioning adenoma; TSA, transsphenoidal adenectomy; ENT, ear, nose and throat; CSF, cerebrospinal fluid.

Results are displayed in **Table 2** and summarized in **Figure 3**. Univariable and multivariable analyses with confounders applied for each determinant can be found in **Supplementary Table S1**. None of the demographic factors were associated with total costs. Preoperative factors associated with higher costs were patients with RCC (B: 9201; 95% CI: 1173, 17230) compared to NFA, macroadenoma (B: 4627; 95% CI 210, 9044) or giant adenoma (B: 19106; 95% CI 12336, 25877) compared to microadenoma. Preoperative panhypopituitarism (B: 5750; 95% CI 1052, 10447), reduced visual acuity (B: 6048; 95% CI 2537, 9560) and third ventricle invasion (B: 14613; 95% CI 7613, 21613) were also associated with increased costs, compared to patients without these characteristics. Patients with pituitary adenoma apoplexy were related to lower costs (B: -27404; 95% CI -36081, -18728) compared to patients without apoplexy. Finally, patients with dependent functional status were associated with higher costs (B: 12231; 95% CI 3985, 20477) compared to functionally independent patients, while none of the comorbidities was related to increased costs.

**Table 2:** Univariable and multivariable linear regression analyses displaying significant determinants of total costs.

Pre-operative factors* Demography, disease characteristics and comorbidities*													
			Univariable analyses					Multivariable analyses					
Variable		Groups	Beta		95% CI for B		P-value	Beta	95% CI for B				P-value
					Lower bound	Upper bound			Lower bound			Upper bound	
**Clinical diagnosis** **(reference: NFA)**	RCC		8301		300	16301	0,042	9201	1173			17230	**0,025**
**Tumour size** **(reference: micro)**	Macroadenoma		5134		1091	9177	0,013	4627	210			9044	**0,040**
	Giant adenoma		17363		10723	24002	<0,001	19106	12336			25877	**<0,001**
**Dependent functional status** **(reference: functionally independent)**	Functional status		13012		4920	21104	0,002	12231	3985			20477	**0,004**
**Hypopituitarism** **(reference: no hypopituitarism)**	Panhypopituitarism		10175		5303	15047	<0,001	5750	1052			10447	**0,017**
**Vision loss** **(incl. VFD)**			8104		4929	11278	<0,001	6048	2537			9560	**0,001**
**Invasion third ventricle N=253**			24667		18734	30600	<0,001	14613	7613			21613	**<0,001**
**Apoplexy N=241**			582		-4246	5410	0,812	-27404	-36081			-18728	**<0,001**
**Operative factors** ***Surgical objective and approach***													
**Year of surgery** **(reference: 2015)**	2016			8608	3886	13330	<0,001	4858	2013			7702	**0,001**
	2018			1407	-3023	5837	0,532	3191	536			5845	**0,019**
**Emergency surgery** **(reference: elective surgery)**	Emergency			8474	2727	14220	0,004	10363	1422			19305	**0,023**
**Surgery indication** **(reference: prevention of disease progression)**	Visual field deficit			7248	1065	13430	0,022	6924	1168			12681	**0,019**
	Vision loss			9276	2771	15780	0,005	6987	890			13083	**0,025**
	Hydrocephalus			57723	45006	70440	<0,001	37534	23787			51282	**<0,001**
**Surgery aim** **(reference: GTR)**	Emergency			12363	6473	18252	<0,001	18719	8357			29080	**<0,001**
	Debulking			*7404*	*-127*	*14936*	*0,054*	8993	2003			15984	**0,012**
**Surgery intended effect** **(reference: remission)**	Reduce medication dosage (adenomas)			7437	2447	12427	0,004	7407	2132			12681	**0,006**
	Recover hydrocephalus			56990	45209	68772	<0,001	49111	34934			63287	**<0,001**
**Surgical procedure** **(reference: endoscopic TSA)**	Endoscopic transventricular			5734	-11129	22597	0,504	-17200	-32486			-1914	**0,028**
	Combined transsphenoidal and transcranial			39816	30716	48915	<0,001	38494	29191			47797	**<0,001**
**OR time in minutes**				81	73	89	<0,001	62	50			74	**<0,001**
**Intradural TSA**				32612	26921	38302	<0,001	17128	10421			23836	**<0,001**
**Surgery performed by two neurosurgeons and ENT-surgeon** **(reference: surgery performed by two neurosurgeons)**				14248	10139	18358	<0,001	11532	7440			15623	**<0,001**
**Intra-operative CSF leak**				10706	7506	13907	<0,001	5565	2620			8509	**<0,001**
**Reconstruction with nasoseptal flap** **(reference: reconstruction without nasoseptal flap)**				16483	12495	20470	<0,001	6812	2582			11043	**0,002**
**Post-operative factors** ***Complications and recovery***													
**Complications**	Intracerebral haemorrhage or hematoma requiring intervention			17902	9084	26720	<0,001	12077		4468		19687	**0,002**
	Postoperative CSF leak			23347	18589	28105	<0,001	14232		9667		18797	**<0,001**
	Hypothalamic injury			93580	69224	117937	<0,001	68770		45630		91909	**<0,001**
	Thromboembolic event			18742	7920	29563	0,001	8423		1813		15032	**0,013**
**New hypopituitarism** **(reference: no new hypopituitarism)**	New panhypopituitarism			19883	10522	29244	<0,001	12633		4183		21082	**0,004**
**Diabetes insipidus classification** **(reference: no diabetes insipidus)**	3: DI between 2 weeks and 6 months			10945	5314	16575	<0,001	9213		4126		14300	**<0,001**
	4: persistent DI >6 months			17844	11680	24007	<0,001	11765		5873		17656	**<0,001**
**CSF leak treatment** **(reference: ELD) N=26**	Surgical intervention			25906	-1127	52940	0,060	26098		1786		50411	**0,037**
**LOS of index hospitalization in days**				1652	1506	1799	<0,001	1331		1139		1523	**<0,001**
**Admission at high care unit during index hospitalization**				20511	14461	26560	<0,001	12154		6413		17895	**<0,001**
**LOS at high care unit during index hospitalization in days** **N=18**				9493	7800	11186	<0,001	9200		2135		16265	**0,015**
**Recovery**													
**Visible tumour residual on MRI (reference=no residual)** **N=255**				7025	3679	10370	<0,001	3577		162	6992		**0,040**
**Recovery hypersecretion <6 months (reference=yes)** **N=124**	No			7634	3954	11314	<0,001	3723		148	7298		**0,041**
**Recovery vision loss <6 months. (reference=yes)** **N=66**	No			18096	3398	32794	0,017	30833		12998	48669		**0,001**

B, unstandardized regression coefficient; n/a, not applicable; N, number; NFA, non-functioning adenoma; FSH, follicle stimulating hormone; RCC, Rathke’s cleft cyst; COPD, chronic obstructive pulmonary disease; mFI, modified frailty index; VFD, visual field deficit; CSF, cerebrospinal fluid; GTR, gross total resection; OR, operating room; ENT, ear, nose and throat; DI, diabetes insipidus; SIADH, syndrome of inappropriate antidiuretic hormone secretion; ELD, external lumbar drain; LOS, length of stay.

Bold indicates statistically significant p-values. If preferred by the journal’s style, bold can be changed to regular text.

Surgical factors associated with higher costs were emergency surgery, e.g. for hydrocephalus and reduced visual acuity, (B: 10363; 95% CI 1422, 19305) compared to elective surgery. One additional minute in OR was associated with a €62 increase in costs (95% CI 50, 74). Surgery indications VFD (B: 6924; 95% CI 1168, 12681), reduced visual acuity (B: 6987; 95% CI 890, 13083) or hydrocephalus (B: 37534; 95% CI 23787, 51282) were associated with increased costs, compared to a more elective indication or preventive surgery (e.g. growing mass close to the optic chiasm without VFD yet). In reference to GTR as surgical aim, emergency surgery (B: 18719; 95% CI 8357, 29080) and debulking (B: 8993; 95% CI 2003, 15984) were also related to higher costs. When the intended effect of surgery was to lower medication dosage or alleviate hydrocephalus, costs were €7407 (95% CI 2132, 12681) and €49111 (95% CI 34934, 63287) higher than achieving remission, respectively. Compared to endoscopic trans-sphenoidal surgery, combined transsphenoidal/transcranial (B: 38494; 95% CI 29191, 47797) and intradural surgery (B: 17128; 95% CI 10421, 23836) were associated with higher costs, whereas endoscopic transventricular surgery was related to lower costs (B: -17200; 95% CI -32486, -1914). Furthermore, surgery performed by two neurosurgeons and ENT surgeon together (B: 11532; 95% CI 7440, 15623) compared to two neurosurgeons, intra-operative CSF leak (B: 5565; 95% CI 2620, 8509) and skull base reconstruction with nasoseptal flap (B: 6812; 95% CI 2582, 11043) compared to reconstruction methods without nasoseptal flap (e.g., with abdominal fat, fibrin glue, free mucosa graft and/or fascia lata) were associated with higher costs.

As expected, LOS in days (B: 1331; 95% CI 1139, 1523), high care unit admission (B: 12154; 95% CI 6413, 17895) and LOS in days at the high care unit (B: 9200; 95% CI 2135, 16265) were associated with higher cost. Complications including intracerebral haemorrhage or hematoma (B: 12077; 95% CI 4468, 19687), postoperative CSF leak (B: 14232; 95% CI 9667, 18797), hypothalamic injury (B: 68770; 95% CI 45630, 91909), thromboembolic event (B: 8423; 95% CI 1813, 15032), new panhypopituitarism (B: 12633; 95% CI 4183, 21082) and diabetes insipidus class 3 (B: 9213; 95% CI 4126, 14300) or 4 (B: 11765; 95% CI 5873, 17656) compared to no diabetes insipidus were also related to higher costs. Of patients with postoperative CSF leak, surgical treatment (B: 26098; 95% CI 1786, 50411) was associated with higher costs compared to external lumbar drain (ELD) placement. Surprisingly, LOS was shorter in patients with ELD compared to surgically treated patients (data not shown). Visiting the emergency department or being readmitted within 30 days after surgery were not significantly related to increased costs. Patients with persistent hypersecretion (B: 3723; 95% CI 148, 7298), reduced visual acuity (B: 30833; 95% CI 12998, 48669) or a visible tumour residual on MRI (B: 3577; 95% CI 162, 6992) were also associated with higher costs compared to remission of hormonal hypersecretion, normalisation of visual acuity and complete resection of tumour mass.

We performed additional multivariable analyses to compare the effect of cost outliers on the association between clinical diagnosis and total costs. We excluded cases with >€80.000 (n=3) and this changed the association of clinical diagnoses and total costs for patients with RCC (B: 7081; 95% CI -4347, 7976), craniopharyngioma (B: 7080; 95% CI 1887, 12274) and Cushing’s disease (B: 5672; 95% CI 1455, 9890) (data not shown).

Based on cost determinants, we analysed total costs, OR duration and LOS of patients with uneventful or complex course. Uneventful cases were defined as patients with micro- or macroadenoma undergoing elective TSA performed by two neurosurgeons, without (post)operative complications and a mean LOS of ≤ 3 days. Likewise, patients were complex if at least one cost determinant was present (e.g., patients with giant adenoma, emergency surgery, tumours invading the third ventricle) (**Supplemental Table S2** and **Supplemental Figure S3**). In the total cohort, patients with uneventful and complicated course had mean costs of €8879 (3210) and €17551 (14250), respectively. For patients with NFA, costs were €9059 (3911) for uneventful cases and €16190 (12778) for complex cases. Total costs were €6766 for one uneventful case with RCC, while all other patients with RCC or craniopharyngioma had a complicated course with mean total costs €33157 (23170). Mean costs of specific cost domains, OR duration and LOS are provided in **Supplemental Table S2**.

## 4 Discussion

This study evaluated the in-hospital direct costs (€16380) for the year after surgery, with higher mean costs in patients with RCCs (€23263) and craniopharyngiomas (€36129) compared to pituitary adenomas (€14462). In line with previous studies, total costs were mostly attributable to direct costs of the surgical procedure (55%) and to a lesser extent to hospitalization (28%), irradiation, and diagnostic investigations or consultations (17%) (14, 42, 43). Additionally, we identified factors associated with higher total costs, that are generally well explained by more complex patient and tumour characteristics, or a more complex clinical course (**Figure 3**). So, increased costs were logically related to healthcare utilization (e.g., longer OR time, LOS, ICU), in concurrence with previous reports (5, 14–16, 24, 28, 44–50). Additionally, we found that patients with RCCs, dependent functional status, tumours invading the third ventricle, specific surgical approaches (i.e., combined transsphenoidal/transcranial, intradural, involvement of ENT-surgeon, nasoseptal flap skull base reconstruction) were increased costs. In patients with postoperative CSF leak, we found that surgical re-intervention was particularly related to higher cost compared with ELD placement.

Interestingly, we found that patients with apoplexy had lower costs. We attempted to address this finding by exploring differences in patients’ characteristics. In patients with apoplexy, we found that mean OR time was 30 min shorter compared to the total cohort, but other cost-driving factors (i.e. surgical approach, LOS and complications) did not explain lower costs. Thus, the explanation for lower costs in patients with apoplexy remains speculative, e.g. shared follow-up with regional centre, incomplete diagnostic workup preoperatively. It is also important to note that higher costs in patients undergoing emergency surgery are not attributable to apoplexy, but to patients having CSF leak or hydrocephalus.

### 4.1 General Relevance and Implications

Costs evaluations are necessary to improve care paths in line with the VBHC approach. Structural changes can be made within healthcare trajectories to limit the use of expensive or unnecessary interventions that do not improve outcome or add value for patients (e.g., prolonged LOS in select patients). Moreover, cost determinants can be used as proxy for healthcare utilization. Therefore, insights in costs enable physicians to consider costs and outcomes simultaneously in clinical decision making and make more individualized paths. For the first time, this study provides a costs baseline, since reference data for European patients with pituitary tumours was lacking, and enables us to identify cost-drivers, relate costs to clinical outcomes and detect changes in cost over time. By doing so, we can evaluate the effectiveness of future value-improving initiatives during different parts of the care trajectory.

### 4.2 Healthcare Costs in Perspective

It is difficult to compare our outcomes to those of earlier studies, because of different healthcare systems and cost assessment methods. Economic evaluations for patients with pituitary tumours undergoing surgery have been mainly performed in the USA (12–16, 42–45, 48), reporting costs ranging from $20000 to $35000 (14, 23, 42, 51). However, the USA has a different healthcare system than most European countries (52). European economic evaluations, reported lower annual direct costs of €8000-12000 (17, 29, 53) for patients with acromegaly (17, 18, 29, 54), €2000 for prolactinoma (20) and €3000 (19) for NFA. However, these studies included yearly chronic care instead of the year of surgical intervention associated with a peak in costs (18–21, 26). Nevertheless, surgery may be cost-effective in the long-term for patients with acromegaly or prolactinoma compared to pharmacotherapy, particularly when remission is achieved since costs of drugs will decrease after intervention (29, 55, 56).

### 4.3 Differences With Other Studies

In contrast to previous studies, we did not find significant associations between costs and age (19), smoking status (14, 47), comorbidities (23, 28, 47), MFI-5 score (51), Cushing’s disease (24, 44, 45) or readmissions (44, 45). Age was not related to higher costs (19), which may be explained by the higher proportion of complex cases at younger age, compared to more patients with NFAs at higher age. Together, our results indicate that pituitary tumour characteristics particularly contribute to increased costs, while patient characteristics influence costs only to a lesser extent. Therefore, stratifying care paths based on tumour characteristics may be suitable for improving VBHC.

### 4.4 Implications for VBHC Initiatives

The previously mentioned cost determinants can be used for value-improving initiatives. However, not all determinants may reduce costs effectively, because they are not subject to preventive measures or extremely rare. For example, preoperative panhypopituitarism may be associated with higher costs, but preventing panhypopituitarism is already an important treatment objective. Regardless, these unmodifiable determinants may be used as a sign of more complex disease course and may be relevant in-patient counselling. Likewise, though hypothalamic injury was the strongest cost-driver, this complication was present in only one case and therefore care adjustments likely have a low impact on overall healthcare costs.

#### 4.4.1 LOS

LOS is a clear cost driver of in-hospital costs (24, 45) with an €1331 increase in costs per day. We previously evaluated the effect of reducing LOS to 2-3 days in selected patients and showed that reducing LOS was safe with no significant decrease in costs (32). However, this analysis was based on a small number of patients and used a different cost-analysis methodology. In the current study, using clinical practice data of a large group of patients, we do find that a decrease of LOS indeed reduces costs. In analogy, other studies reported significant cost reduction after reducing LOS to 1-2 days after brain tumour surgery (57–59). Cost data reported in this study will serve as reference point to evaluate the cost-effectiveness of further LOS reduction in select patients.

#### 4.4.2 Functionally Dependent Patients

Higher costs in functionally dependent patients are likely explained by longer LOS and more complications after surgery (60–62). Despite the risk, patients may benefit from brain tumour surgery and recover functional independence (60). Therefore, the risks and benefits of surgery and alternative treatment options should be carefully weighed, while considering individual values. Prehabilitation programs have been designed to improve functional status and general health prior to elective surgery and have shown improved outcomes (63–69). However, these studies focussed on thoracic, abdominal and orthopaedic surgery and therefore results may not be applicable to pituitary surgery patients. Besides, the cost-effectiveness of such interventions remains unclear (70, 71). In the current cohort the total effect of prehabilitation on costs is likely limited, due to the small proportion of functional dependent patients (n=11, 4%).

#### 4.4.3 Tumours Related to the Third Ventricle, RCCs and Craniopharyngiomas

Some unmodifiable determinants of cost are tumours related to the third ventricle, RCCs and craniopharyngiomas, as they are associated with more complications. These patients showed higher, more varying costs, OR time and LOS (**Supplemental Table S2**), possibly reflecting more complex disease course, unpredictable outcomes and more complications (72, 73). Though preferred surgical approach is debated, an experienced surgeon and team is needed for optimal outcomes, but still complications are frequently seen (74). Opting for GTR may be risky, as surrounding structures may be damaged and hypopituitarism may occur. However, subtotal resection may lead to recurrences. As illustrated by our results, complex tumours, extensive surgical approaches and complications are highly prevalent in patients with RCC or craniopharyngioma compared to pituitary adenomas, indicating that a separate treatment trajectory for these patients may be justified. Careful preoperative planning is mandatory and additional imaging techniques could aid choosing the best surgical approach and improve outcomes (75–77). Furthermore, this trajectory may incorporate an earlier postoperative MRI to assess residual tumour volume or recurrence and provide counselling concerning increased risk for panhypopituitarism. Future studies are needed to explain more specifically why patients with RCC or craniopharyngioma are associated with higher costs and which factors increase risks for complications, so that care pathways can be adjusted accordingly.

#### 4.4.4 Postoperative CSF Leak

Identifying patients at risk for postoperative CSF leak and implementing preventive measures likely improves outcomes and reduce costs, thereby improving value for patients and allocating resources (i.e., labour, OR capacity) more efficiently. Postoperative CSF leak was related to over €14000 additional costs per case and occurred in 9,6% and 7,5% in the total cohort or in patients with pituitary adenoma, respectively. Compared to previous studies reporting a prevalence between 0,9 and 5,2% (14, 15, 48, 78, 79) after transsphenoidal pituitary surgery, the prevalence of postoperative CSF leak in the present study was high. However, this needs to be placed in perspective with the complexity of our case-mix in a tertiary referral centre and potential selection bias (i.e., more severe cases undergo surgical intervention rather than ELD placement). Known risk groups for postoperative CSF leak are patients with higher BMI, third ventricle invasion, craniopharyngioma, previous skull base irradiation, prior surgery and intra-operative CSF leak (74, 78, 80–82). For these patients particularly, tailored skull base reconstruction methods are critical to prevent and/or manage CSF leak optimally (83). Also, preventive ELD placement may result in more efficient use of resources and indirectly lower costs, however it will also increase LOS in short-stay protocols. As illustrated by our results, more extensive reconstruction methods (i.e., using nasoseptal flap reconstruction) and ELD placement are costly interventions. However, they may be cost effective when CSF leak or additional surgical intervention is prevented (48, 74, 81, 84).

### 4.5 Strengths and Limitations

Despite previous cost evaluations, this study is to our knowledge the first European study providing a comprehensive overview of the cost of perioperative healthcare for patients with pituitary tumours in a tertiary referral centre. Though, this study has several limitations. First, this study is retrospective, with all inherent limitations. Another limitation is the single centre-nature, with our centre being a tertiary referral centre and both nationally and internationally endorsed pituitary expertise centre, receiving referrals from throughout the country, including more complex cases (e.g., more macroprolactinoma and RCC). Hence, accurate assessment of healthcare usage is subject to hospital registration data, which differs in different hospitals. Consequently, differences in costs between Dutch tertiary referral centres may be partially accountable to the quality of in-hospital costs registration.

Limitations in our costs assessment were that we did not correct for inflation, which may have confounded our cost results across the years. Also, we only included direct costs incurred at our institution, thereby neglecting indirect (e.g., administrative) costs and costs incurred at other institutions. Finally, the costs for medication were not included in this analysis since these are not included in hospital costs. This likely results in underestimating the costs of patients receiving healthcare at other facilities or pharmacological treatment (e.g., patients with functioning adenoma or growth hormone replacement therapy). We encourage future studies to adhere to cost evaluation guidelines to promote interpretability of results (37). Cost-evaluations including costs for medical treatment and hormone replacement therapy are warranted to pursue a good evaluation of management strategies. A limitation in our statistical analyses using pairwise deletion instead of multiple imputation for missing data, which may have biased our results.

It is also important to point out that this study focussed on perioperative healthcare, thereby disregarding the costs of the preoperative trajectory and follow-up care after one year. During of the preoperative care different factors might be profound contributors to higher costs. For example, functional imaging for Cushing’s disease or frequent consultations in prolactinoma patients might be associated with higher costs in preoperative healthcare. Finally, we did not exclude patients with inordinately high costs due to a complicated course which possibly confounded the effect size of some determinants. The two most expensive patients in our cohort costed over €100.000 each. They both presented with hydrocephalus and had a complicated course with surgically treated postoperative CSF leak. One of these patients had a NFA and also experienced intracerebral haemorrhage, multiple organ failure, several infections and hypothalamic damage and was admitted for 69 days. The other patient was functionally dependent, and surgery was complicated by phlebitis and hematemesis secondary to stage IV oesophagitis, being admitted for 44 days. Though we attempted to alleviate confounding effects in the multivariable analyses, we cannot preclude these patients confounded the effect sizes of some cost determinants (hydrocephalus, dependent functional status, surgical treatment of CSF leak, cerebral haemorrhage and hypothalamic syndrome), and as shown in analyses with or without cost outliers, cost outliers influence the significance of some cost determinants (i.e., Cushing’s disease, RCC and craniopharyngioma) (data not shown).

## 5 Conclusion

Insight in healthcare costs and their determinants is necessary to curtail costs and facilitate VBHC initiatives. The present study provides a concise overview of costs and its determinants, which will serve as reference point for future value-improving initiatives and as a proxy for resource utilization. With this information, future studies can investigate costs and outcomes more specifically (e.g., predict complications), so that healthcare pathways can be adjusted strategically according to the VBHC framework. For example, differentiated healthcare pathways (**Figure 3**) for patients with uncomplicated disease course and predictable costs or patients with a complicated course (i.e., presence of one or more cost determinants) and higher, more varying costs. Consequently, developing value-improving initiatives for patients with giant tumours, non-adenomatous aetiology, dependent functional status, third ventricle invasion or extensive surgical approaches may result in reduced costs and improved outcomes. Also, further reduction of LOS seems viable and safe in select patients. In this way, we endeavour to improve value-based healthcare for patients with pituitary tumours.

## Data Availability Statement

The raw data supporting the conclusions of this article will be made available by the authors, without undue reservation.

## Ethics Statement

The studies involving human participants were reviewed and approved by LUMC medical ethics committee, local study number G19·011. Written informed consent for participation was not required for this study in accordance with the national legislation and the institutional requirements.

## Author Contributions

AD, FV, and EH collected the data, and AD and FV conducted the data analysis. The study was performed under supervision of NB. AD and FV wrote the primary version of the manuscript. AZ, MV, AP, WF, and NB supervised the data analysis, contributed to the interpretation of the results, and reviewed and revised the manuscript. All authors contributed to the article and approved the submitted version.

## Conflict of Interest

The authors declare that the research was conducted in the absence of any commercial or financial relationships that could be construed as a potential conflict of interest.

## Publisher’s Note

All claims expressed in this article are solely those of the authors and do not necessarily represent those of their affiliated organizations, or those of the publisher, the editors and the reviewers. Any product that may be evaluated in this article, or claim that may be made by its manufacturer, is not guaranteed or endorsed by the publisher.
