# Supplementary material for: Costs and Its Determinants in Pituitary Tumour Surgery
Source: Front Endocrinol (Lausanne). 2022 Jul 7;13:905019. doi: 10.3389/fendo.2022.905019 (PMC9302462; doi:10.3389/fendo.2022.905019)
Supplement: Supplementary file 1 [file DataSheet_1.pdf]

Supplementary Material

1 Index supplemental material

- **Supplemental table S1**: all univariable and multivariable analyses with confounders specified.
- **Supplemental table S2**: mean total costs, operating room time and length of stay for patients with uneventful or complicated course, including the distribution of total costs into cost categories.
- **Supplemental figure S3**: healthcare trajectory and costs of patients with uneventful or complicated NFA.
- **Supplemental figure S4**: general representation of pituitary tumour healthcare for patients with uneventful or complex course, distinguishing preoperative, perioperative and postoperative care.

**S1: univariate and multivariable linear regression analyses with correction for relevant confounders.**										
**Pre-operative factors**<br>***Demography, disease characteristics and comorbidities***										
		**Univariate analyses**					**Multivariable analyses**			
			**95% CI for B**					**95% CI for B**		
**Variable**	**Groups**	**Beta**	**Lower bound**	**Upper bound**	**P-value**	**Confounders**	**Beta**	**Lower bound**	**Upper bound**	**P-value**
**Sex (reference: male)**	Female	-1481	-4740	1779	0,372	Age	-1229	-4499	2042	0,460
**Age (years)**		74	-21	169	0,125	Clinical diagnosis	10	-91	110	0,850
**BMI**		58	-274	390	0,731	Age, sex	33	-300	366	0,846
**Currently smoking**		2686	-1612	6983	0,220	Age, sex	2870	-1430	7171	0,190
**Clinical diagnosis (reference: NFA)**	Prolactinoma	-3301	-7788	1186	0,149	Sex, tumour size, previous medical treatment	104	-5772	5980	0,972
	FSH-adenoma	-365	-14566	13837	0,960	Size	-8551	-22294	5191	0,222
	Thyrotropinoma	-6212	-20414	7990	0,390	Size	-3158	-16642	10326	0,645
	Acromegaly	113	-4252	4477	0,959	Size, hypertension, congestive heart failure, diabetes mellitus	2278	-2036	6592	0,299
	Cushing's disease	1007	-3618	5633	0,668	Sex, size, hypertension, diabetes mellitus, previous surgical treatment, post-operative cortisol deficiency or panhypopituitarism	5613	-166	11393	*0,057*
	RCC	8301	300	16301	**0,042**	CS invasion	9201	1173	17230	**0,025**
	Craniopharyngioma	21260	15386	27134	**<0,001**	Size, third ventricle invasion, CS invasion, previous surgical treatment,<br>intradural surgery	5130	-1908	12167	0,152
**Tumour size (reference: micro adenoma)**	Macroadenoma	5134	1091	9177	**0,013**	Sex, age, clinical diagnosis	4627	210	9044	**0,040**
	Giant adenoma	17363	10723	24002	**<0,001**	^	19106	12336	25877	**<0,001**
	No clear adenoma	2172	-6341	10685	0,616	^	-1069	-9583	7444	0,805
**Comorbidities**	Hypertension	812	-2752	4376	0,654	Age, BMI, currently smoking, acromegaly, Cushing's disease, thromboembolic event	338	-3635	4310	0,867
	Diabetes Mellitus	-3474	-9183	2235	0,232	Age, BMI, acromegaly, Cushing's disease, thromboembolic event	-4387	-10235	1460	0,141

	Congestive heart failure	-9341	-36140	17458	0,493	Age, BMI, acromegaly, Cushing’s disease, thromboembolic event	-9241	-35697	17215	0,492
	COPD	1074	-14469	16617	0,892	Currently smoking	597	-14988	16183	0,940
	Functional status	13012	4920	21104	**0,002**	Age	12231	3985	20477	**0,004**
**mFI-5 score (reference: mFI-5 score=0)**	1	3433	-181	7046	*0,063*	Age, BMI, currently smoking, acromegaly, Cushing’s disease	3568	-101	7237	*0,057*
	2	-3167	-10334	4001	0,385	^	-2165	-9204	4873	0,545
	3	-3928	-22869	15013	0,683	^	-2706	-20013	14601	0,758
	4	12120	-14591	38830	0,372	^	-1756	-28656	25143	0,898
	5	n/a					n/a			
**Hypopituitarism (reference: no hypopituitarism)**	Hypopituitarism non-corticotrope	-514	-4341	3312	0,791	Clinical diagnosis, size, apoplexy, previous surgical treatment, previous radiation therapy	-443	-3919	3034	0,802
	Cortisol deficiency	2095	-3351	7542	0,449	^	1639	-3580	6857	0,537
	Panhypopituitarism	10175	5303	15047	**<0,001**	^	5750	1052	10447	**0,017**
**Vision loss (incl. VFD)**		8104	4929	11278	**<0,001**	Clinical diagnosis, size, apoplexy	6048	2537	9560	**0,001**
**Cranial nerve palsy**		1042	-5487	7572	0,754	Clinical diagnosis, apoplexy, CS invasion	812	-6810	8434	0,834
**Invasion third ventricle (N=253)**		24667	18734	30600	**<0,001**	Craniopharyngioma, size	14613	7613	21613	**<0,001**
**Invasion cavernous sinus (adenomas, N=241)**		3478	117	6838	**0,043**	Clinical diagnosis, size	1507	-1805	4819	0,371
**Apoplexy (adenomas, N=241)**		582	-4246	5410	0,812	Clinical diagnosis, emergency	-27404	-36081	-18728	**<0,001**
**Prior treatment (reference: no prior treatment)**	Pharmacological (adenomas, N=241)	-1278	-4033	1477	0,362	Prolactinoma, acromegaly, Cushing’s disease	-435	-4191	3321	0,820
	Surgery	1161	-2967	5289	0,580	NFA, Cushing’s disease, craniopharyngioma	-196	-4074	3681	0,921
	Irradiation	-5613	-24602	13377	0,561	Clinical diagnosis	-15842	-33291	1606	*0,075*

**Surgery related factors**<br>***Surgical objective and approach***										
		**Univariate analyses**					**Multivariable analyses**			
			95% CI for B					95% CI for B		
		Beta	Lower bound	Upper bound	P-value		Beta	Lower bound	Upper bound	P-value
**Year of surgery (reference: 2015)**	**2016**	8608	3886	13330	**<0,001**	LOS	4858	2013	7702	**0,001**
	2017	1107	-3528	5742	0,639	LOS	1182	-1591	3954	0,402
	2018	1407	-3023	5837	0,532	LOS	3191	536	5845	**0,019**
**Emergency surgery (reference: elective surgery)**	Emergency	8474	2727	14220	**0,004**	Age, sex, clinical diagnosis, hypertension, diabetes mellitus, congestive heart failure, COPD, apoplexy, third ventricle invasion, hydrocephalus, vision loss	17383	6289	28477	**0,002**
**Surgical procedure (reference: endoscopic TSA)**	Craniotomy	4067	-12796	20931	0,635	Age, sex, clinical diagnosis, size, hypertension, diabetes mellitus, congestive heart failure, COPD	6542	-8263	21347	0,385
	Endoscopic transventricular	5734	-11129	22597	0,504	Age, sex, clinical diagnosis, size, hypertension, diabetes mellitus, congestive heart failure, COPD	-17200	-32486	-1914	**0,028**
	Combined transsphenoidal and transcranial	39816	30716	48915	**<0,001**	Age, sex, clinical diagnosis, size, hypertension, diabetes mellitus, congestive heart failure, COPD	38494	29191	47797	**0,000**
**Surgery indication (reference: prevention of disease progression)**	Hypersecretion (adenomas)	2629	-1573	6831	0,219	Age, sex, clinical diagnosis, hypertension, diabetes mellitus, congestive heart failure, COPD, vision loss or VFD, emergency	1359	-6407	9124	0,731
	Growth of tumour residual	3339	-5735	12412	0,469	Age, sex, clinical diagnosis, hypertension, diabetes mellitus, congestive heart failure, COPD	3231	-5317	11779	0,457
	Apoplexy (adenomas)	4340	-997	9678	0,110	Age, sex, clinical diagnosis, size, hypertension, diabetes mellitus, congestive heart failure, COPD	4190	-1094	9473	0,120
	Visual field deficit	7248	1065	13430	**0,022**	Age, sex, clinical diagnosis, size, hypertension, diabetes	6924	1168	12681	**0,019**

						mellitus, congestive heart failure, COPD				
	Vision loss	9276	2771	15780	**0,005**	Age, sex, clinical diagnosis, size, hypertension, diabetes mellitus, congestive heart failure, COPD	6987	890	13083	**0,025**
	Hydrocephalus	57723	45006	70440	**0,000**	Age, sex, clinical diagnosis, size, hypertension, diabetes mellitus, congestive heart failure, COPD, third ventricle invasion	37534	23787	51282	**<0,001**
	CSF leak	11329	-5818	28477	0,194	Age, sex, clinical diagnosis, size, hypertension, diabetes mellitus, congestive heart failure, COPD, third ventricle invasion, previous pharmacological treatment	17510	-475	35495	*0,056*
	Cranial nerve deficit (adenomas)	-158	-11651	11335	0,978	Age, sex, clinical diagnosis, hypertension, diabetes mellitus, congestive heart failure, COPD, SC invasion, apoplexy	-1566	-12897	9766	0,786
	Headache	-4440	-18787	9907	0,543	Age, sex, clinical diagnosis, hypertension, diabetes mellitus, congestive heart failure, COPD, SC-invasion, apoplexy, hydrocephalus	-5979	-19554	7596	0,386
	Nose congestion	-2401	-26038	21235	0,842	Age, sex, clinical diagnosis, hypertension, diabetes mellitus, congestive heart failure, COPD	4329	-18592	27249	0,710
	Behavioural alterations	-3300	-26936	20337	0,784	Age, sex, clinical diagnosis, hypertension, diabetes mellitus, congestive heart failure, COPD	-20357	-43328	2613	*0,082*
	Biopsy	9112	-14525	32748	0,448	Age, sex, clinical diagnosis, hypertension, diabetes mellitus, congestive heart failure, COPD	7052	-17280	31384	0,569
**Surgery aim (reference: GTR)**	Decompression	7486	3993	10979	**<0,001**	Age, sex, clinical diagnosis, size, hypertension, diabetes mellitus, congestive heart failure, COPD, SC-invasion, vision loss or VFD	3055	-3390	9500	0,351

	Emergency	12363	6473	18252	**<0,001**	Age, sex, clinical diagnosis, hypertension, diabetes mellitus, congestive heart failure, COPD, apoplexy, hydrocephalus	18719	8357	29080	**<0,001**
	Exploration	3647	-3046	10341	0,284	Age, sex, clinical diagnosis, hypertension, diabetes mellitus, congestive heart failure, COPD	611	-6674	7896	0,869
	Debulking	7404	-127	14936	*0,054*	Age, sex, clinical diagnosis, hypertension, diabetes mellitus, congestive heart failure, COPD	8993	2003	15984	**0,012**
	Biopsy	2620	-15695	20935	0,778	Age, sex, clinical diagnosis, hypertension, diabetes mellitus, congestive heart failure, COPD	-5789	-23159	11580	0,512
**Surgery intended effect (reference: remission)**	Protect or recover vision	5931	2848	9015	**<0,001**	Age, sex, clinical diagnosis, hypertension, diabetes mellitus, congestive heart failure, COPD, vision loss of VFD	7616	-1336	16567	*0,095*
	Medication intolerance (adenomas, N=241)	7437	2447	12427	**0,004**	Age, sex, clinical diagnosis, size, hypertension, diabetes mellitus, congestive heart failure, COPD	7407	2132	12681	**0,006**
	Reduce sudden headache/vision loss	2585	-3331	8501	0,390	Age, sex, clinical diagnosis, hypertension, diabetes mellitus, congestive heart failure, COPD, apoplexy, hydrocephalus	9956	-7825	27737	0,271
	Recover hydrocephalus	56990	45209	68772	**<0,001**	Age, sex, clinical diagnosis, hypertension, diabetes mellitus, congestive heart failure, COPD, third ventricle invasion	49111	34934	63287	**<0,001**
	Recover CN function (adenomas, N=241)	-1913	-18413	14588	0,820	Age, sex, clinical diagnosis, size, hypertension, diabetes mellitus, congestive heart failure, COPD, apoplexy, CS invasion	3532	-14006	21070	0,692
	Recover CSF	10597	-5904	27097	0,207	Age, sex, clinical diagnosis, hypertension, diabetes mellitus, congestive heart failure, COPD, previous pharmacological treatment	14786	-1036	30608	*0,067*

	Improve nasal congestion	-3134	-26355	20087	0,791	Age, sex, clinical diagnosis, hypertension, diabetes mellitus, congestive heart failure, COPD	11675	-12446	35797	0,341
	PA diagnosis	2173	-14327	18674	0,796	Age, sex, clinical diagnosis, hypertension, diabetes mellitus, congestive heart failure, COPD	-2266	-18649	14117	0,785
	Enable irradiation	16622	-6599	39843	0,160	Age, sex, clinical diagnosis, hypertension, diabetes mellitus, congestive heart failure, COPD	13515	-8654	35685	0,231
	Improve pituitary function	-5360	-28581	17860	0,650	Age, sex, clinical diagnosis, hypertension, diabetes mellitus, congestive heart failure, COPD, vision loss or VFD	4791	-18478	28060	0,685
	Reduce headache	-5079	-21579	11422	0,545	Age, sex, clinical diagnosis, size, hypertension, diabetes mellitus, congestive heart failure, COPD	-4659	-22480	13162	0,607
**OR time in minutes**		81	73	89	**0,000**	Age, sex, clinical diagnosis, size, hypertension, diabetes mellitus, congestive heart failure, COPD, third ventricle invasion, CS invasion, extended approach, intradural approach, ENT surgeon, intra-operative CSF leak	**62**	**50**	**74**	**0,000**
**Extended approach**		25156	20024	30288	**0,000**	Age, sex, clinical diagnosis, size, hypertension, diabetes mellitus, congestive heart failure, COPD, third ventricle invasion, CS invasion, OR time, intra-operative CSF leak, post-operative CSF leak	2184	-2665	7033	0,376
**Intradural surgery**		32612	26921	38302	**<0,001**	Age, sex, clinical diagnosis, size, hypertension, diabetes mellitus, congestive heart failure, COPD, third ventricle invasion, intra-operative CSF leak, post-operative CSF leak	17128	10421	23836	**<0,001**
**Surgery performed with**		14248	10139	18358	**<0,001**	Age, sex, clinical diagnosis, hypertension, diabetes mellitus,	11532	7440	15623	**<0,001**

**additional ENT-surgeon (reference: surgery by two neurosurgeons)**						congestive heart failure, COPD, previous surgical resection				
**Intra-operative CSF leak**		10706	7506	13907	**<0,001**	Age, sex, clinical diagnosis, size, hypertension, diabetes mellitus, congestive heart failure, COPD, intradural surgery	5565	2620	8509	**<0,001**
**Reconstruction with nasoseptal flap (reference: other reconstruction methods)**		16483	12495	20470	**<0,001**	Age, sex, clinical diagnosis, hypertension, diabetes mellitus, congestive heart failure, COPD, extended approach, intradural surgery, intra-operative CSF leak	6812	2582	11043	**0,002**

**Post-operative factors** *Complications and recovery*										
			95% CI for B					95% CI for B		
**Variable**	Groups	Beta	Lower bound	Upper bound	P-value	Adjusted for	Beta	Lower bound	Upper bound	P-value
**Complications**	Intracerebral haemorrhage or hematoma requiring intervention	17902	9084	26720	**<0,001**	Age, size, clinical diagnosis, third ventricle invasion, extended approach, previous radiation therapy	12077	4468	19687	**0,002**
	Postoperative CSF leak	23347	18589	28105	**<0,001**	Age, sex, BMI, RCC, craniopharyngioma , size, third ventricle invasion, extended approach, intradural surgery	14232	9667	18797	**<0,001**
	Hyponatremia (including SIADH)	5277	1453	9102	**0,007**	Age, sex, BMI, emergency department visit <30 days, readmission <30 days, LOS	-172	-2892	2548	0,901
	Hypothalamic injury	93580	69224	117937	**<0,001**	Age, craniopharyngioma, size, third ventricle invasion, intracranial haemorrhage	68770	45630	91909	**<0,001**
	Thromboembolic event	18742	7920	29563	**0,001**	Age, hypertension, diabetes mellitus, congestive heart failure, LOS	8423	1813	15032	**0,013**

	Epistaxis requiring intervention	2482	-3881	8844	0,443	Age, size, hypertension, diabetes mellitus, congestive heart failure, ENT-surgeon	906	-4896	6708	0,759
	Vision deterioration	-2931	-21945	16082	0,762	Age, intracranial haemorrhage	-2661	-21148	15826	0,777
	New cranial nerve palsy	n/a				Age, CS invasion	n/a			
**New hypopituitarism (reference: no new hypopituitarism)**	New hypopituitarism (excl. cortisol deficiency)	-8544	-34660	17572	0,520	Age, craniopharyngioma, size	-3536	-26871	19799	0,766
	New cortisol deficiency	6756	-2604	16117	0,156	Age, Cushing's disease, craniopharyngioma, size	4343	-4040	12725	0,309
	New panhypopituitarism	19883	10522	29244	**<0,001**	Age, Cushing's disease, craniopharyngioma, size	12633	4183	21082	**0,004**
**Diabetes insipidus classification (reference: no DI)**	1: transient DI <48 hours, spontaneously resolving without treatment	3903	-1617	9423	0,165	RCC, craniopharyngioma, size	3660	-1282	8601	0,146
	2: transient DI <2 weeks	4644	-501	9789	*0,077*	RCC, craniopharyngioma, size	4405	-251	9061	*0,064*
	3: DI between 2 weeks and 6 months	10945	5314	16575	**<0,001**	RCC, craniopharyngioma, size	9213	4126	14300	**<0,001**
	4: persistent DI >6 months	17844	11680	24007	**<0,001**	RCC, craniopharyngioma, size	11765	5873	17656	**<0,001**
**CSF leak treatment (reference: ELD) N=26**	Surgery	25906	-1127	52940	*0,060*	Year of surgery	26098	1786	50411	**0,037**
	ELD and surgery	25506	-3693	54706	*0,084*	Year of surgery	27731	-71	55534	*0,051*
**LOS of index hospitalization in days**		1652	1506	1799	**<0,001**	Age, clinical diagnosis, functional status, hydrocephalus, intracerebral haemorrhage, CSF leak, thromboembolic event, hypothalamus damage, new panhypopituitarism	1331	1139	1523	**<0,001**
**Admission at high car unit during index hospitalization**		20511	14461	26560	**<0,001**	Hydrocephalus, hypothalamic injury, haemorrhage	12154	6413	17895	**<0,001**

**LOS at high care unit during index hospitalization in days N=18**		9493	7800	11186	**<0,001**	Hydrocephalus, hypothalamic injury, haemorrhage	9200	2135	16265	**0,015**
**Emergency department visit <30 days**		1127	-3720	5975	0,647	Hyponatremia/SIADH, DI, epistaxis	-499	-4913	3914	0,824
**Readmission <30 days**		7302	2849	11755	**0,001**	Emergency department visit <30 days, CSF leak, hyponatremia/SIADH	2375	-2562	7313	0,344
**Evaluation at 6 months**										
**Visible tumour residual on MRI (reference: no tumour residual) N=255**		7025	3679	10370	**<0,001**	Clinical diagnosis, size, CS invasion, decompression, debulking	3577	162	6992	**0,040**
**Recovery hypersecretion <6 months (reference: remission) N=124**	Partial	5348	486	10210	**0,031**	Size, GTR	3093	-1335	7521	0,169
	No	7634	3954	11314	**<0,001**	Size, GTR	3723	148	7298	**0,041**
**Recovery hypopituitarism <6 months (reference: recovered) N=137**	Partial	4254	-6184	14693	0,422	Size, GTR	1290	-9301	11881	0,810
	No	6139	-100	12378	*0,054*	Size, GTR	3448	-2991	9886	0,291
**Recovery VFD <6 months (reference: normalisation) N=114**	Partial	3802	-2416	10020	0,228	Size, GTR	1322	-5265	7909	0,692
	No	5982	-6004	17968	0,325	Size, GTR	7297	-5020	19615	0,243
**Recovery vision loss <6 months. (reference: normalisation) N=66**	Partial	-1500	-12347	9347	0,783	Size, GTR	-4080	-14710	6550	0,446
	No	18096	3398	32794	**0,017**	Size, GTR	30833	12998	48669	**0,001**

**Recovery cranial nerve palsy <6 months (reference: recovered) N=22**	Partial	-1164	-13581	11252	0,847	Size, GTR	-335	-11579	10910	0,951
	No	n/a				Size, GTR	n/a			

Abbreviations: *B* unstandardized regression coefficient, *n/a* not applicable, *N* number, *BMI* body mass index, *NFA* non-functioning adenoma, *FSH* follicle stimulating hormone, *RCC* Rathke’s cleft cyst, *COPD* chronic obstructive pulmonary disease, *mFI* modified frailty index, *VFD* visual field deficit, *CSF* cerebrospinal fluid, *GTR* gross total resection, *OR* operating room, *ENT* ear, nose and throat, *DI* diabetes insipidus, *SIADH* syndrome of inappropriate antidiuretic hormone secretion, *ELD* external lumbar drain, *LOS* length of stay, *ICU* intensive care unit

Table S2: Mean costs, OR time and LOS in patients with uneventful* or complicated disease course. Note that patients with uncomplicated disease course show lower mean costs, OR duration and LOS, with smaller standard deviation. Of all patients with uneventful disease course, all but one patient was diagnosed with pituitary adenoma. With the exception of one case with RCC, all patients with RCC or craniopharyngioma had a complicated course.

		**Total costs**			**Surgery costs**			**Hospitalization costs**			**Diagnostic procedures or consultations**			**OR time**			**LOS**		
		Mean	SD	N	Mean	SD	N	Mean	SD	N	Mean	SD	N	Mean	SD	N	Mean	SD	N
**Total cohort**	Uncomplicated	8879	3210	37	5199	2352	37	1731	638	37	1948	1010	37	131	41	37	2	1	37
	Complicated	17551	14250	232	9574	9013	232	5044	5295	232	2922	1619	232	199	137	232	6	7	232
**Pituitary adenomas**	Uncomplicated	8937	3235	36	5244	2369	36	1730	647	36	1962	1020	36	133	41	36	2	0	36
	Complicated	15409	11021	204	8087	7047	204	4532	4313	204	2779	1491	204	186	125	204	6	6	204
**NFA**	Uncomplicated	9059	3911	22	5523	2852	22	1698	716	22	1838	856	22	134	48	22	2	0	22
	Complicated	16190	12778	96	8414	7799	96	4848	5434	96	2919	1512	96	187	131	96	6	8	96
**Prolactinoma**	Uncomplicated	8329	914	6	4558	927	6	1636	297	6	2135	1090	6	125	26	6	3	1	6
	Complicated	12147	6151	33	7118	5062	33	2921	1297	33	2107	968	33	150	54	33	4	2	33
**Rathke's cleft cyst, craniopharyngioma**	Uncomplicated	6766	0	1	3569	0	1	1773	0	1	1424	0	1	90	0	1	3	0	1
	Complicated	33157	23170	28	20413	13531	28	8775	9144	28	3961	2102	28	290	183	28	11	12	28

*Uneventful cases are defined as patients with micro- or macroadenoma undergoing elective TSA performed by two neurosurgeons, with LOS ≤ 3 days and without (post)operative complications.

Figure S3: Examples of care trajectories of patients with NFA with uneventful* or complicated disease course, showing lower costs and smaller standard deviation in patients with uneventful compared to complex disease course. Specific data on mean costs, OR duration and LOS are displayed in table S2.

NFA with <u>uneventful</u> care trajectory: mean total costs are 9059 ± 3911.

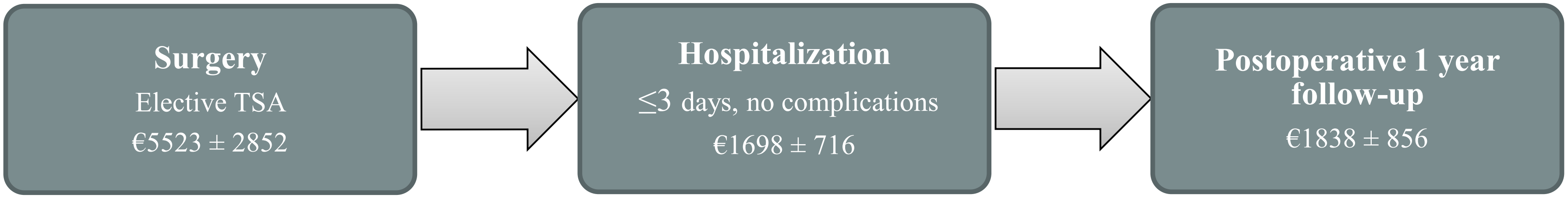

NFA with <u>complicated</u> care trajectory: mean total costs of 16190 ± 12778.

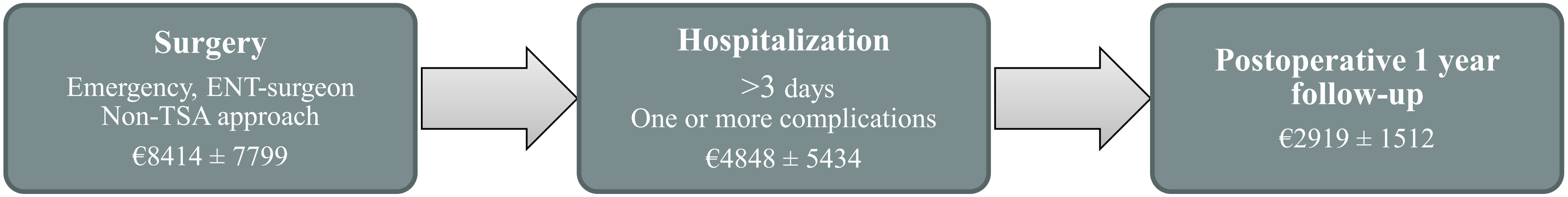

*Uneventful cases are defined as patients with micro- or macroadenoma undergoing elective TSA performed by two neurosurgeons, with LOS ≤ 3 days and without (post)operative complications.

Supplemental figure S4: general representation of pituitary tumour healthcare for patients with uneventful or complex course, separated by preoperative, perioperative and postoperative care.

Reference care pathway

Combined outpatient clinic consultation with endocrinologist and neurosurgeon

Imaging: MRI and CT

General and endocrinological bloodwork

TSA with two neurosurgeons

Hospitalization for 2-3 days

6m evaluation with MRI

Neurosurgical and endocrinological workup

Diagnostic procedures → Diagnosis, shared decsion making → **Admission and surgical treatment** → Remission, complications → **Post-operative workup**

Complex care pathway

Complex nasal-sphenoidal anatomy or previous surgery
- ➔ ENT consultation

Relation with optic chiasm or nerve
- ➔ Ophthalmologist consultation

Inconclusive MRI/CT
- ➔ MET/PET, FET/PET

Hypopituitarism
- ➔ Additional endocrinological consultations

Recurrent tumour, RCC

Supra-regional/international referrals
- ➔ Administrative complexity

Paediatric
- ➔ Paediatrician consultation

Emergency surgery (e.g., acute decompression of nerve(s), hydrocephalus or apoplexy)

Complex nasal-sphenoidal anatomy or previous surgery
- ➔ Surgery performed by two neurosurgeons and ENT surgeon

Large and/or invasive tumours
- ➔ Longer OR time
- ➔ Alterative surgical approach: extended TSA, intradural TSA, craniotomy (combined with TSA)

Significant comorbidities and/or complications
- ➔ Prolonged or ICU hospitalization
- ➔ Additional diagnostic and therapeutic procedures

Frail and/or functionally dependent elderly
- ➔ Prolonged hospitalization (depending on social support system at home)

New hypopituitarism
- ➔ Extensive endocrinological workup and individualised HRT

Tumour residual and/or no endocrinological remission
- ➔ Additional (nuclear) imaging
- ➔ Adjuvant treatment (reoperation, pharmacological, irradiation)

Vision loss
- ➔ Ophthalmological follow-up

(New) sino-nasal morbidity (e.g., anosmia)
- ➔ ENT follow-up
